# Spontaneous large-scale autolysis in *Clostridium acetobutylicum* contributes to generation of more spores

**DOI:** 10.3389/fmicb.2015.00950

**Published:** 2015-09-09

**Authors:** Zhen Liu, Kai Qiao, Lei Tian, Quan Zhang, Zi-Yong Liu, Fu-Li Li

**Affiliations:** ^1^Key Laboratory of Biofuels and Shandong Provincial Key Laboratory of Energy Genetics, Qingdao Institute of Bioenergy and Bioprocess Technology – Chinese Academy of SciencesQingdao, China; ^2^Sinopec Fushun Research Institute of Petroleum and PetrochemicalsFushun, China

**Keywords:** autolysis, sporulation, *Clostridium acetobutylicum*, ClosTron, autolysin

## Abstract

Autolysis is a widespread phenomenon in bacteria. In batch fermentation of *Clostridium acetobutylicum* ATCC 824, there is a spontaneous large-scale autolysis phenomenon with significant decrease of cell density immediately after exponential phase. To unravel the role of autolysis, an autolysin-coding gene, CA_C0554, was disrupted by using ClosTron system to obtain the mutant *C. acetobutylicum lyc*::int(72). The lower final cell density and faster cell density decrease rate of *C. acetobutylicum* ATCC 824 than those of *C. acetobutylicum lyc*::int(72) indicates that CA_C0554 was an important but not the sole autolysin-coding gene responding for the large-scale autolysis. Similar glucose utilization and solvents production but obvious lower cell density of *C. acetobutylicum* ATCC 824 comparing to *C. acetobutylicum lyc*::int(72) suggests that lysed *C. acetobutylicum* ATCC 824 cells were metabolic inactive. On the contrary, the spore density of *C. acetobutylicum* ATCC 824 is 26.1% higher than that of *C. acetobutylicum lyc*::int(72) in the final culture broth of batch fermentation. We speculated that spontaneous autolysis of metabolic-inactive cells provided nutrients for the sporulating cells. The present study suggests that one important biological role of spontaneous large-scale autolysis in *C. acetobutylicum* ATCC 824 batch fermentation is contributing to generation of more spores during sporulation.

## Introduction

Autolysis is a widespread phenomenon in bacteria such as *Bacillus subtilis* (Lacriola et al., 2013), *Escherichia coli* (Leduc and Heijenoort, 1980), *Lactobacillus helveticus* (Lortal et al., 1997). Autolysis of *B. subtilis* growing cells could be induced by adding chloramphenicol, which caused a significant decrease of OD_590__nm_ in 420 min (Chung et al., 2009). Addition of cephaloridine could induce the autolysis of *E. coli* grown at 37°C in rich medium, OD_600__nm_ decreased over 80% in 40 min (Leduc et al., 1982). On the other hand, spontaneous autolysis has been reported in *Helicobacter pylori* and *Propionibacterium freudenreichii* (Lemée et al., 1995; Fujita et al., 2005). In batch fermentation of *H. pylori* KZ109, OD_600__nm_ decreased 66% in 12 h (Fujita et al., 2005). In batch fermentation of *P. freudenreichii* CNRZ 725, up to 88% of OD_650__nm_ decrease was observed after maximal growth (Lemée et al., 1995). Comparing to *B. subtilis* and *E. coli*, addition of inducts was not needed and there were no obvious stationary phase in the growth curve of bacteria with spontaneous autolysis.

Spontaneous autolysis phenomenon was also found in *Clostridium acetobutylicum*. In batch fermentation of *C. acetobutylicum* ATCC 824, the OD_620__nm_ decreased more than 60% in 15 h immediately after exponential phase (Barber et al., 1979). In batch fermentation of *C. acetobutylicum* P262, the total cell counts decreased 50% from 35 to 60 h of fermentation (Van der Westhuizen et al., 1982). Minier et al. (1990) carried out *C. acetobutylicum* batch fermentation, using mineral ultrafiltration membranes to confine the microbial cells in the fermenter while the permeate was subject to heat treatment (70°C for 20 min) before recycling. Under this condition, the activity of autolysins in the fermentation broth was strongly depressed, leading to twofold increase of the maximum OD_620__nm_ (Minier et al., 1990), indicating that autolysins played important roles in the spontaneous autolysis of *C. acetobutylicum* growing cells.

Autolysins are enzymes that can break down the cell wall of microorganisms (Smith et al., 2000) and have been found in many bacteria, such as *E. coli* (Heidrich et al., 2001), *B. subtilis* (Blackman et al., 1998), *Enterococcus faecalis* (Mesnage et al., 2008), *C. acetobutylicum* (Croux and García, 1991). The known physiological functions of autolysins include regulation of cell growth, enlargement of the peptidoglycan sacculus, peptidoglycan turnover, production of signaling molecules, recycling of peptidoglycan turnover products, cell separation during cell division, assembly of secretion systems, developmental lysis, biofilm formation (Vollmer et al., 2008). Two enzymes with lytic activity have been purified from the supernatant of *C. acetobutylicum* ATCC 824 and identified as glycoprotein and amidase, respectively (Webster et al., 1981; García et al., 1988). An extracellular lytic enzyme was purified and characterized as muramidase by Croux et al. (1992) in the same species. Furthermore, two autolysin-coding genes, CA_C0554 (Croux and García, 1991) and SMB_G3117 (Yang et al., 2013), have been characterized in *C. acetobutylicum*, thereinto CA_C0554 has been functionally verified by expression in *E. coli* (Croux and García, 1992).

Since spontaneous autolysis usually leads to significant loss of cells, it is interesting to understand the biological role behind this phenomenon. Spontaneous autolysis of *H. pylori* cells accompanies release of intracellular proteins into the extracellular space, and the intact cells receive the released proteins on the cell surface in order to survive of the adverse environment (Fujita et al., 2005). Though several autolysins have been characterized in *C. acetobutylicum*, the biological role of spontaneous autolysis in this species is unclear until now. In this study, CA_C0554, one gene contributing to autolysis, was disrupted to study the role of autolysin on the significant decrease of cell density, and to investigate the biological role of spontaneous large-scale autolysis in *C. acetobutylicum* ATCC 824.

## Materials and Methods

### Bacterial Strains and Cultivation Conditions

All strains and plasmids used in this study are listed in **Table 1**. *E. coli* was cultivated aerobically at 37°C in Luria-Bertani medium containing per liter 10 g tryptone, 5 g yeast extract, 10 g NaCl. Where indicated, appropriate antibiotics were added (chloramphenicol 25 μg ml^-1^ or tecracycline 10 μg ml^-1^).

**Table 1:** Strains and plasmids used in this study.

Strains or plasmids	Relevant characteristics	Reference or source
**Strains**		
*Clostridium acetobutylicum* ATCC 824	Wild type	American type culture collection
*C. acetobutylicum lyc*::int(72)	Group II intron inserted at 72/73 bp of *lyc* (CA_C0554), Erm^R^	This study
*Escherichia coli* DH5α	F^-^, Φ80*lac*ZΔM15, Δ(*lac*ZYA), recA1, endA1, *hsd*R17 (r_k_^-^, m_k_^+^), *pho*A, *sup*E44 *thi*1, *gyr*A96, *rel*A1, ^-^	Grant et al. (1990)
**Plasmids**		
pAN2	*Φ3T*I, p15A origin, Tet^R^	Heap et al. (2007)
pMTL007C-E2	*ltrA, Ll.ltrB* intron, pCB102, ColE1 origin, Cm^R^	Heap et al. (2010)
pMTL007-*lyc*	pMTL007C-E2 re-targeted for *lyc*	This study

*Clostridium acetobutylicum* ATCC 824 or its mutant was cultivated anaerobically at 37°C. During the mutagenesis process, clostridial growth medium was used, which contains per liter 0.75 g KH_2_PO_4_, 0.75 g K_2_HPO_4_, 0.71 g MgSO_4_⋅7H_2_O, 0.01 g MnSO_4_⋅7H_2_O, 0.01 g FeSO_4_⋅7H_2_O, 1 g NaCl, 2 g (NH_4_)_2_SO_4_, 5 g yeast extract, and 2 g asparagine, the pH was adjusted to 6.6 with NH_4_OH, and glucose was added at a final concentration of 2.5 g l^-1^ after autoclaving (Roos et al., 1985). While in fermentation, P2 medium (Baer et al., 1987) containing per liter 60 g glucose, 0.5 g K_2_HPO_4_, 0.5 g KH_2_PO_4_, 2.2 g CH_3_COONH_4_, 0.2 g MgSO_4_⋅7H_2_O, 0.01 g MnSO_4_⋅H_2_O, 0.01 g NaCl, 0.01 g FeSO_4_⋅7H_2_O, 1 mg ρ-aminobenzoic acid, 1 mg thiamine, 0.01 mg biotin was used. As an anaerobic indicator, resazurin was added into the clostridial growth medium and P2 medium at a concentration of 1 mg l^-1^. Where indicated, appropriate antibiotics (e.g., 15 μg ml^-1^ thiamphenicol or 2.5 μg ml^-1^ erythromycin) were added in the medium.

### Construction of *C. acetobutylicum* lyc::int(72)

Generation of the 1,4-β-*N*-acetylmuramidase (coded by CA_C0554) negative mutant *C. acetobutylicum lyc*::int(72) was carried out by using the ClosTron system (Heap et al., 2007; Tan et al., 2015). PCR primers for the disruption of the *lyc* gene (CA_C0554) were designed by using the freely available TargeTron tool at http://www.clostron.com (Perutka et al., 2004). The primers used in this study are listed in **Table 2**. For retargeting of the intron to CA_C0554, splicing by overlap extension PCR (Warrens et al., 1997) was carried out using lyc72/73s-IBS, lyc72/73s-EBS1d, lyc72/73s-EBS2 and EBS universal primers (**Table 2**) with a template made by mixing two plasmids pMTL20IT1 and pMTL20IT2 in a ratio of 1:1 (Heap et al., 2010). The purified PCR product was ligated to pEASY^TM^-E1 and verified by DNA sequencing. After digestion with *Hin*dIII and *Bsp*1407I, the verified fragment was ligated into *Hin*dIII/*Bsp*1407I-restricted pMTL007C-E2. The correct ligation was confirmed by colony (grown on CGM plate containing 25 μg ml^-1^ chloramphenicol) PCR using the primers spofdx-seq-F1 and pMTL007-R1 (**Table 2**) with the 548 bp product.

**Table 2:** Primers used in this study.

Primer name	Sequence (5′-3′)
lyc72/73s-IBS	AAAAAAGCTTATAATTATCCTTAAGTGGCGTAGAAGTGCGCCCAGATAGGGTG
lyc72/73s-EBS1d	CAGATTGTACAAATGTGGTGATAACAGATAAGTCGTAGAAGTTAACTTACCTTTCTTTGT
lyc72/73s-EBS2	TGAACGCAAGTTTCTAATTTCGATTCCACTTCGATAGAGGAAAGTGTCT
EBS-universal	CGAAATTAGAAACTTGCGTTCAGTAAAC
spofdx-seq-F1	GATGTAGATAGGATAATAGAATCCATAGAAAATATAGG
pMTL007-R1	AGGGTATCCCCAGTTAGTGTTAAGTCTTGG
up-intron	GAAAACCTTTGTGAACAGCTGAAAA
down-intron	TCGATACTTTGACCTAATGTTACTT
southern-intron-R	GAAAGTATAGGAACTTCACGCGTCG

The retargeted plasmid was transformed into *E. coli* DH5α together with pAN2 for *in vivo* methylation (Grant et al., 1990). The methylated plasmid was electroporated into *C. acetobutylicum* ATCC 824 and selected on the CGM plates containing 15 μg ml^-1^ thiamphenicol. Verification of correct transformants was carried out by colony PCR using the primers spofdx-seq-F1 and pMTL007-R1 with the 548 bp product. The correct transformants were resuspended in CGM medium and spread onto the CGM plates containing 2.5 μg ml^-1^ erythromycin. Correct integrants were confirmed by colony PCR using the primers up-intron and down-intron (**Table 2**) with the ∼2200 bp product, or using the primers up-intron and EBS universal with the 451 bp product. The correct integrant was passaged at least six times on the non-selective medium to lose the ClosTron plasmid *lyc*-retargeted pMTL007C-E2.

Southern blot analysis was performed to screen the integrants containing a single intron insertion. A *lyc* intron specific probe (518 bp) was obtained by PCR using primers lyc72/73s-EBS2 and southern-intron-R (**Table 2**) with genome of an integrant as the template. Genomic DNA of the wild strain and integrants was digested by *Hin*dIII, separated in 0.8% agarose gel, then transferred onto a nylon membrane. The hybridization was carried out at 44°C overnight. After incubation with anti-Dig-AP conjugate, the blot was visualized with NBT/BCIP.

### Fermentation

Batch fermentation of *C. acetobutylicum* ATCC 824 or its mutant was carried out in 250-ml bottles containing 50 ml of the P2 medium anaerobically at 37°C. Fresh seeds (5%) were inoculated to start the fermentation. The initial glucose concentration, OD_600__nm_ and pH were 80 g l^-1^, 0.05-0.1 and 7.0, respectively. Cell-free supernatant samples were stored at -20°C for further analyses.

### Spore Density Determination

At the end of fermentation, 100 μl of culture broth was sampled, stayed at 80°C for 10 min to inactivate the vegetative cells (Lehmann et al., 2012), diluted to the 10^-1^, 10^-2^, 10^-3^, 10^-4^, 10^-5^, 10^-6^, and 10^-7^ fold of the original concentration. The diluted samples were spread onto the P2 plates without antibiotics and incubated at 37°C overnight. The plates with colonies’ number between 100 and 600 were selected to count. The spore density was calculated as follows:

Spore density = Colonies’ number × Dilution fold/100 μl.

### Analytical Methods

The optical density (OD) was measured at 600 nm using a 2600 spectrophotometer (Unico Instruments CO., Ltd., Shanghai, China).

The concentration of glucose was determined by high performance liquid chromatography with an Agilent 1260 series, equipped with a Bio-Rad Aminex HI-Plex H column (300 mm × 7.7 mm) and a refractive index detector. Operating conditions were as follows: mobile phase, 5 mM H_2_SO_4_; flow rate, 0.6 ml min^-1^; and column temperature, 65°C.

Acetone, butanol and ethanol were all detected by a GC system (Agilent 7890B) equipped with a flame ionization detector and a column (HP-INNOWAX 19091N-213, 30 m length, 0.32 mm inner diameter). The column temperature was raised from 35 to 45°C at a rate of 2°C min^-1^, then to 150°C at a rate of 40°C min^-1^, after which it was maintained at 150°C for 1 min. The injector and detector temperature were 200 and 250°C, respectively. Samples were extracted with threefold volume of ethyl acetate containing ioamyl alcohol as the internal standard, then 1 μl of the organic phase was injected.

## Results

### Construction of *C. acetobutylicum* lyc::int(72)

**FIGURE 1:**
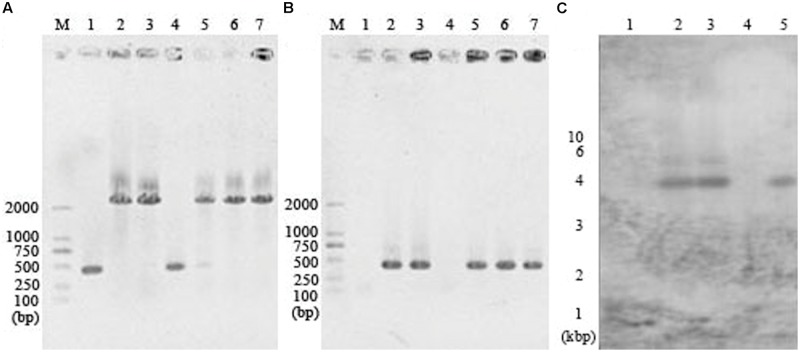
**Confirmation of the CA_C0554 disruption mutant *C. acetobutylicum lyc*::int(72) by colony PCR and southern blot analysis.** Screening of putative integrants was performed by colony PCR with primers up-intron and down-intron **(A)** or primers up-intron and EBS universal **(B)**. M, marker; 1, *C. acetobutylicum* ATCC 824; 2-7, erythromycin-resistant clones. Verification of the correct integrants with a single intron insertion was carried out by southern blot analysis **(C)**. M, marker; 1, *C. acetobutylicum* ATCC 824; 2-5, putative integrants screened by colony PCR.

CA_C0554, one of the autolysin-coding genes found in *C. acetobutylicum* ATCC 824, was disrupted by ClosTron system to investigate the role of autolysin on the spontaneous large-scale autolysis. Putative integrants were screened by colony PCR from the erythromycin-resistant colonies. Using primers up-intron and down-intron, a product of ∼2200 bp was obtained with a putative integrant as template, while a product of 425 bp was obtained with the wild strain as template (**Figure 1A**). Using primers up-intron and EBS universal, a product of 451 bp was obtained with a putative integrant as template, while no product was obtained with the wild strain as template (**Figure 1B**). Five putative integrants were screened and four of them were verified by southern blot analysis with a *lyc* intron specific probe. As shown in **Figure 1C**, the integrant in lane 5 with only one hybridized belt was verified as the correct CA_C0554 disrupted mutant and named as *C. acetobutylicum lyc*::int(72).

### Effect of CA_C0554 Disruption on Metabolism

**FIGURE 2:**
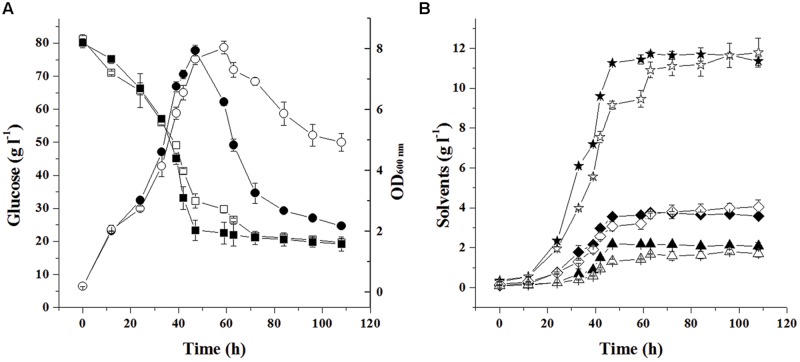
**Time course of batch fermentation with glucose as substrate by *C. acetobutylicum* ATCC 824 and *C. acetobutylicum lyc*::int(72).** ATCC 824-glucose (■), ATCC 824-OD (•), ATCC 824-butanol (★), ATCC 824-acetone (⧫), ATCC 824-ethanol (▲), *lyc*::int(72)-glucose (□), *lyc*::int(72)-OD_600_ nm (○), *lyc*::int(72)-butanol (☆), *lyc*::int(72)-acetone (◇), *lyc*::int(72)-ethanol (△). Data are presented as the average ± standard deviation values of three parallel replicates.

The effect of CA_C0554 disruption was investigated. Firstly, batch fermentation of *C. acetobutylicum* ATCC 824 and *C. acetobutylicum lyc*::int(72) was compared. As shown in **Figure 2**, during the exponential phase (0-47 h), the cell growth of the 2 strains were similar. In fermentation of *C. acetobutylicum* ATCC 824, OD_600__nm_ decreased 72.5% (7.94 ± 0.18 to 2.18 ± 0.04) from 47 to 108 h, which was similar with the previous reports (Barber et al., 1979; Lehmann and Lütke-Eversloh, 2011; Lehmann et al., 2012). On the other hand, during fermentation of *C. acetobutylicum lyc*::int(72), OD_600__nm_ decreased 35.6% (7.66 ± 0.19 to 4.93 ± 0.30) in the same phase (47–108 h). The OD_600__nm_ decrease rate (0.094 h^-1^) of *C. acetobutylicum* ATCC 824 is 2.09 fold of that (0.045 h^-1^) of. *C. acetobutylicum lyc*::int(72), which means CA_C0554 plays an important role in the cell density decrease in the batch fermentation of *C. acetobutylicum* ATCC 824. On the other hand, since there was still 35.6% decrease of cell density in the batch fermentation of *C. acetobutylicum lyc*::int(72), it seems that besides CA_C0554, there are other autolysins responsible for the cell density decrease in the batch fermentation of *C. acetobutylicum* ATCC 824. In another word, CA_C0554 was an important but not the sole gene coding for autolysins responsible for the spontaneous large-scale autolysis.

In batch fermentation of *C. acetobutylicum* ATCC 824, the consumed glucose was 60.84 ± 0.77 g l^-1^, which was very similar to that (60.45 ± 1.02 g l^-1^) consumed in batch fermentation of *C. acetobutylicum lyc*::int(72). The final concentration of acetone (3.58 ± 0.08 g l^-1^ vs. 4.05 ± 0.36 g l^-1^), butanol (11.36 ± 0.22 g l^-1^ vs. 11.79 ± 0.73 g l^-1^), and ethanol (2.08 ± 0.18 g l^-1^ vs. 1.71 ± 0.18 g l^-1^) were also comparable between the batch fermentation of *C. acetobutylicum* ATCC 824 and that of *C. acetobutylicum lyc*::int(72), respectively. This suggests that the obvious change of cell density did not influence the overall metabolic level of the CA_C0554 disrupted mutant *C. acetobutylicum lyc*::int(72).

### Effect of CA_C0554 Disruption on Sporulation

**FIGURE 3:**
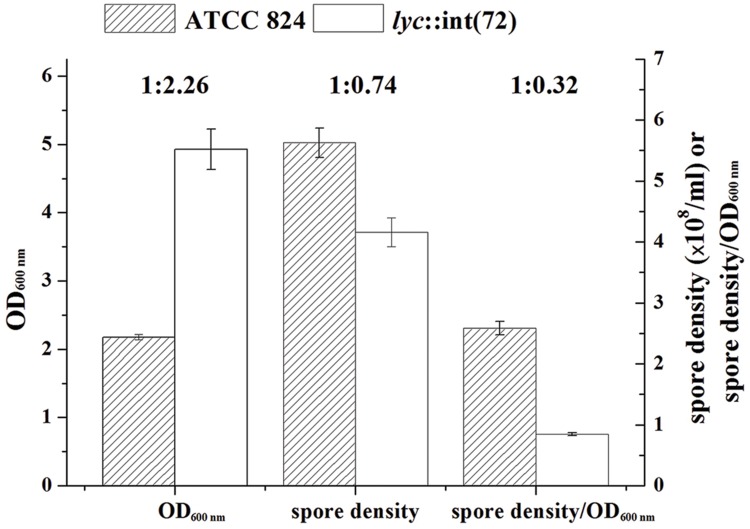
**Comparison of cell density and spore density in final (108 h) culture of batch fermentation of *C. acetobutylicum* ATCC 824 and *C. acetobutylicum lyc*::int(72).** Data are presented as the average ± standard deviation values of three parallel replicates.

To investigate the effect of CA_C0554 disruption on sporulation, the spore density of the final culture in batch fermentation was determined. In the end (108 h) of batch fermentation, the spore density of *C. acetobutylicum lyc*::int(72) (4.16 ± 0.24 × 10^8^/ml) was 26.1% lower than that of *C. acetobutylicum* ATCC 824 (5.63 ± 0.24 × 10^8^/ml; **Figure 3**). On the other hand, the OD_600__nm_ (4.93 ± 0.30) of *C. acetobutylicum lyc*::int(72) at 108 h was 2.26 fold of that (2.18 ± 0.04) of *C. acetobutylicum* ATCC 824 (**Figure 3**). OD_600__nm_ represents the whole biomass including metabolic-inactive cells, vegetative cells and spores, hence the ratio of spore density to OD_600__nm_ could represent the ratio of spores in the whole biomass. As shown in **Figure 3**, the ratio of spore density to OD_600__nm_ of *C. acetobutylicum lyc*::int(72) at 108 h was 0.32 fold of that of *C. acetobutylicum* ATCC 824. This indicates that the significant decrease of spore ratio in the whole biomass for the mutant was caused by disruption of CA_C0554, an important autolysin-coding gene.

## Discussion

*C. acetobutylicum* ATCC 824 is an anaerobic, Gram-positive strain, which is one of the important strains for biobutanol production (Liu et al., 2010; Tracy et al., 2011; Zhang et al., 2012). As a spore-forming strain, the sporulation process of *C. acetobutylicum* ATCC 824 has been widely investigated in recent years (Bi et al., 2011; Al-Hinai et al., 2014). In this study, an autolysin-coding gene, CA_C0554, was disrupted to study the biological role of spontaneous large-scale autolysis in *C. acetobutylicum* ATCC 824.

In batch fermentation of CA_C0554 disrupted mutant *C. acetobutylicum lyc*::int(72), the OD_600__nm_ decrease rate was only 47.8% of that in *C. acetobutylicum* ATCC 824 batch fermentation. This suggests CA_C0554 was an important but not the sole gene coding for autolysins responsible for the large-scale autolysis. In an earlier study, an autolysis-deficient mutant *C. acetobutylicum lyt-1* has been isolated by ethyl methane sulfonate mutagenesis using *C. acetobutylicum* P262 as parent (Allcock et al., 1981). In *C. acetobutylicum* P262 batch fermentation, 50% OD_600__nm_ decrease has been observed after exponential phase, but no obvious OD_600__nm_ change was found in *C. acetobutylicum lyt-1* batch fermentation (Van der Westhuizen et al., 1982). This was because on one hand *C. acetobutylicum lyt-1* produced less autolysin than the parent P262 strain, which was similar to this study, on the other hand *C. acetobutylicum lyt-1* had an altered cell wall which was more resistant to autolysins (Allcock et al., 1981).

The trends of glucose utilization and solvents (acetone, butanol, and ethanol) production were similar between the batch fermentation of *C. acetobutylicum* ATCC 824 and that of *C. acetobutylicum lyc*::int(72). This suggests that the significant change of cell density did not influence the substrate utilization and products formation of the CA_C0554 disrupted mutant *C. acetobutylicum lyc*::int(72). Similar phenomenon has been reported by Minier et al. (1990), in which autolysins in the broth were subject to heat treatment (70°C for 20 min) before recycling, while cells were confined by mineral ultrafiltration membranes in the batch fermentation of *C. acetobutylicum* ATCC 824.

Since increased cell density did not lead to increased substrate utilization and product formation in mutant *C. acetobutylicum lyc*::int(72), it is speculated that the increased cells in *C. acetobutylicum lyc*::int(72) batch fermentation, namely, the cells lysed by autolysins in *C. acetobutylicum* ATCC 824 batch fermentation were metabolic inactive. It indicated that the spontaneous large-scale autolysis is selective in the batch fermentation of *C. acetobutylicum* ATCC 824, in other words, the cells without metabolic activity were lysed. After disruption of CA_C0554, less metabolic-inactive cells were lysed, meanwhile less spores formed, which suggests the autolysis of metabolic-inactive cells could benefit the sporulation. We speculated that metabolic-inactive cells were lysed by autolysins to release nutrients on which the sporulating cells could feed. A killing factor has been characterized in *B. subtilis*, by which part of cells were lysed to release nutrients that could be fed on by sporulating cells (González-Pastor et al., 2003).

Spore forming bacteria initiate the sporulation process to survive of environments unfavorable for growth, while in the environments suitable for vegetative growth, germination of spores happened (Nicholson et al., 2000; Yang et al., 2009). The more spores, the larger survival probability. Hence during sporulation, *C. acetobutylicum* would form spores as more as possible. Normally, the metabolic-inactive cells are considered useless to the sporulation process. However, a large quantity of metabolic-inactive cells were lysed by autolysins such as the protein coded by CA_C0554. Nutrients released by lysed cells were utilized by sporulating cells. Therefore, providing nutrients for sporulation to form more spores under the unfavorable environment is an important biological role of spontaneous large-scale autolysis in the batch fermentation of *C. acetobutylicum* ATCC 824.

In summary, an important autolysin-coding gene, CA_C0554, was disrupted to obtain *C. acetobutylicum lyc*::int(72), the mutant of *C. acetobutylicum* ATCC 824. In the final culture of batch fermentation, though the OD_600__nm_ of *C. acetobutylicum lyc*::int(72) was 126% higher than that of *C. acetobutylicum* ATCC 824, the utilization of substrate and solvents formation were similar between mutant and wild strain. This suggested that the lysed cells of *C. acetobutylicum* ATCC 824 were metabolic inactive. In addition, the spore density of *C. acetobutylicum* ATCC 824 is 26.1% higher than that of *C. acetobutylicum lyc*::int(72), suggesting the lysed metabolic-inactive cells could provide nutrients for the sporulating cells to form more spores. In conclusion, one important biological role of spontaneous large-scale autolysis in *C. acetobutylicum* ATCC 824 batch fermentation is contributing to generation of more spores during sporulation.

## Conflict of Interest Statement

The authors declare that the research was conducted in the absence of any commercial or financial relationships that could be construed as a potential conflict of interest.
